# A novel double-chain silver(I) coordination polymer: *catena*-poly[[[μ-aqua-aqua­disilver(I)]-bis­(μ_3_-5-methyl­pyrazine-2-carboxyl­ato)] dihydrate]

**DOI:** 10.1107/S160053680802984X

**Published:** 2008-09-24

**Authors:** Bin Zhai, Xiangfei Zhang, Maotian Xu

**Affiliations:** a Department of Chemistry, Shangqiu Normal University, Shangqiu 476000, Henan, People’s Republic of China

## Abstract

In the title silver(I) coordination polymer, {[Ag_2_(C_6_H_5_N_2_O_2_)_2_(H_2_O)_2_]·2H_2_O}_*n*_, the [Ag_2_(μ_2_-H_2_O)(H_2_O)] cores are extended by anti­parallel 5-methyl­pyrazine-2-carboxyl­ate (*L*) ligands, forming a novel double-chain structure. Both Ag^+^ cations show a distorted square-pyramidal coordination. Ag1 is bonded to two water molecules, one *L* N atom, one N atom and one carboxylate O atom from a neighbouring *L*, whereas Ag2 is surrounded by two *L* N atoms, two *L* carboxylate O atoms and one bridging water molecule. O—H⋯O hydrogen-bonding inter­actions involving water clusters and carboxyl­ate O atoms link the mol­ecules into a three-dimensional supra­molecular architecture, which is further consolidated by weak C—H⋯O inter­actions and π–π stacking inter­actions [centroid–centroid distance 3.643 (5) Å].

## Related literature

For related literature, see: Ciurtin *et al.* (2001 ▶, 2003 ▶); Dong *et al.* (2000 ▶); Garribba *et al.* (2006 ▶); Liu *et al.* (2007 ▶); Ptasiewicz-Bak & Leciejewicz (2000 ▶); Shang *et al.* (2007 ▶); Tanase *et al.* (2006 ▶); Etter (1990 ▶).
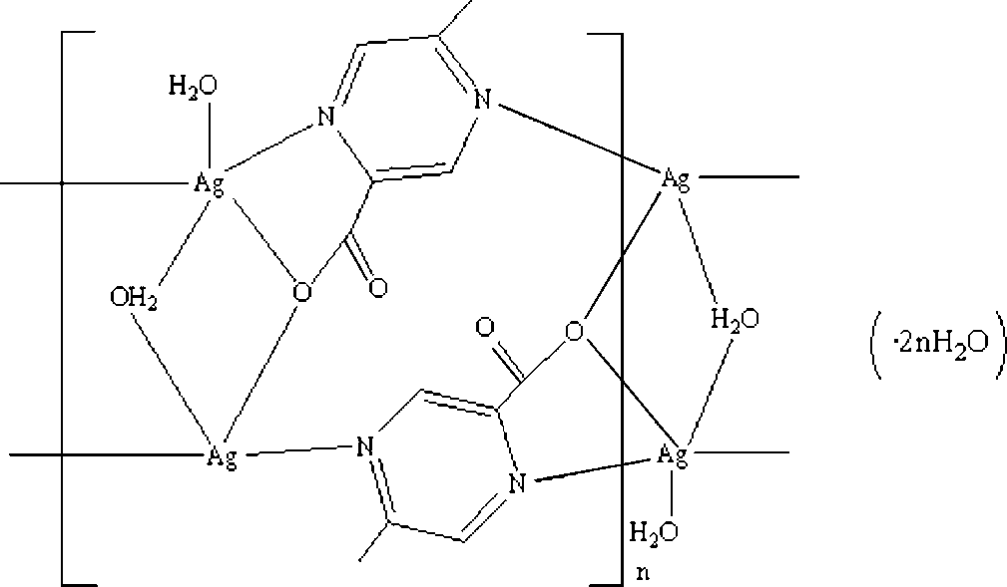


## Experimental

### 

#### Crystal data

[Ag_2_(C_6_H_5_N_2_O_2_)_2_(H_2_O)_2_]·2H_2_O
*M* *_r_* = 562.04
Triclinic, 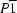

*a* = 6.9481 (5) Å
*b* = 10.1827 (8) Å
*c* = 13.483 (1) Å
α = 107.503 (1)°
β = 100.185 (1)°
γ = 103.164 (1)°
*V* = 854.4 (1) Å^3^
*Z* = 2
Mo *K*α radiation
μ = 2.34 mm^−1^
*T* = 293 (2) K
0.24 × 0.20 × 0.16 mm

#### Data collection

Bruker APEX CCD area-detector diffractometer
Absorption correction: multi-scan (*ABSCOR*; Higashi, 1995 ▶) *T*_min_ = 0.581, *T*_max_ = 0.698
4422 measured reflections
2982 independent reflections
2518 reflections with *I* > 2σ(*I*)
*R*_int_ = 0.014

#### Refinement

*R*[*F* ^2^ > 2σ(*F* ^2^)] = 0.027
*wR*(*F* ^2^) = 0.071
*S* = 1.07
2982 reflections
236 parameters
H-atom parameters constrained
Δρ_max_ = 0.39 e Å^−3^
Δρ_min_ = −0.51 e Å^−3^

### 

Data collection: *SMART* (Bruker, 2001 ▶); cell refinement: *SAINT* (Bruker, 2003 ▶); data reduction: *SAINT*; program(s) used to solve structure: *SHELXS97* (Sheldrick, 2008 ▶); program(s) used to refine structure: *SHELXL97* (Sheldrick, 2008 ▶); molecular graphics: *DIAMOND* (Brandenburg & Putz, 2005 ▶); software used to prepare material for publication: *SHELXTL* (Sheldrick, 2008 ▶).

## Supplementary Material

Crystal structure: contains datablocks global, I. DOI: 10.1107/S160053680802984X/kj2095sup1.cif

Structure factors: contains datablocks I. DOI: 10.1107/S160053680802984X/kj2095Isup2.hkl

Additional supplementary materials:  crystallographic information; 3D view; checkCIF report

## Figures and Tables

**Table 1:** Selected bond lengths (Å)

Ag1—N1	2.260 (3)
Ag1—N3	2.311 (3)
Ag1—O6	2.478 (3)
Ag1—O5	2.517 (3)
Ag1—O4	2.598 (3)
Ag2—N4^i^	2.233 (3)
Ag2—N2	2.250 (3)
Ag2—O2	2.558 (3)
Ag2—O5^ii^	2.688 (3)
Ag2—O4^ii^	2.809 (3)

Symmetry codes: (i) 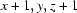; (ii) 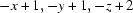.

**Table 2:** Hydrogen-bond geometry (Å, °)

*D*—H⋯*A*	*D*—H	H⋯*A*	*D*⋯*A*	*D*—H⋯*A*
C1—H1⋯O4	0.93	2.38	3.061 (4)	130
O5—H5*B*⋯O2^ii^	0.85	1.94	2.675 (4)	144
O5—H5*A*⋯O7^iii^	0.85	1.91	2.756 (4)	175
O6—H6*A*⋯O7^iv^	0.85	2.00	2.828 (4)	166
O6—H6*B*⋯O1^v^	0.85	1.96	2.794 (4)	168
O7—H7*A*⋯O8^vi^	0.85	1.86	2.698 (4)	168
O7—H7*B*⋯O3^vii^	0.85	1.87	2.712 (4)	171
O8—H8*A*⋯O1	0.85	2.02	2.867 (4)	176
O8—H8*B*⋯O3	0.85	2.13	2.965 (4)	166
O8—H8*B*⋯O4	0.85	2.44	3.116 (4)	137

Symmetry codes: (ii) 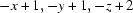; (iii) 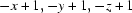; (iv) 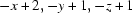; (v) 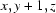; (vi) 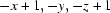; (vii) 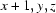.

**Fig. 1.**
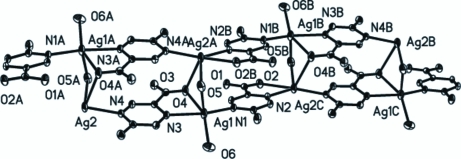
A one-dimensional double chain structure of the title compound comprising the Ag2O2 core. [Symmetry codes: A 2 - x, 1 - y, 1 - z; B 1 - x, 1 - y, -z; C x - 1, y, z - 1.]. Displacement ellipsoids are drawn at the 15% probability level.

**Fig. 2.**
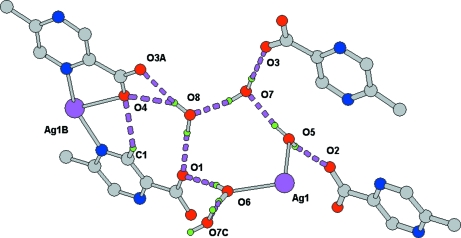
The detailed environment of O—H···O and C—H···O hydrogen bonds interactions.

**Fig. 3.**
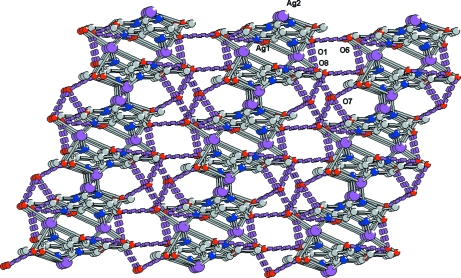
Three-dimensional supramolecular architecture of the title compound. Hydrogen bonds are indicated by dashed lines.

## supplementary crystallographic information

### Comment

During the past two decades poly-carboxylic acid ligands have aroused great interest for chemists because many coordination polymers with this series of ligands have shown intriguing structures and potential applications in the optical, electric and magnetic areas. 5-methylpyrazine-2-carboxylic acid contains N and O donor atoms, which makes it a good building block for constructing functional materials. For example, Dong *et al.* (2000) use Cu(*L*)_2_(H_2_O) as a building block for constructing novel one-dimensional hetero-bimetallic Cu(II)–Ag(I) frameworks. Tanase *et al.* (2006) investigate the magnetic properties of Co(II), Ni(II) and Fe(II) compounds with HL, in structures where *L* is also involved in intricate supramolecular interactions. In this work we describe how using HL and corresponding silver(I) salts under hydrothermal conditions, a novel one-dimensional double silver(I) framework with Ag_2_(µ_2_-H_2_O)(H_2_O) cores can be isolated.

As is shown in Fig. 1, the title compound comprises two crystallographically independent silver(I) atoms, two deprotonated ligands *L*, one bridged coordinated water molecule, one terminal coordinated water molecule and two lattice water molecules. Ag1 is five-coordinated in the square-pyramidal geometry by two coordinated water molecules, one *L* nitrogen atom, one nitrogen atom and one carboxylate oxygen atom from a neighboring *L*. The coordinated water molecule O5 occupies the apical site and the other four atoms occupy the plane with the mean deviation of 0.0463 (1) Å. Ag1 lies above the plane at a distance of 0.3523 (2) Å. Ag2 is also five-coordinated in the square-pyramidal geometry by two *L* nitrogen atoms, two *L* carboxylate oxygen atoms and one bridged water molecule.

There exist two kinds of crystallographically different *L* ligands which make a dihedral angle of 13.786 (2)°. These ligands, in anti-parallel pairs, alternatively link Ag_2_(µ_2_-H_2_O)(H_2_O) cores, forming a novel one-dimensional double chain structure along the crystallographic [101] direction. The distances of Ag2—O5 and Ag2—O4 are longer than other Ag—O distances (Table 1). However all the Ag—N and Ag—O bond distances fall in the normal range.

The formation of this novel framework also reveals great potential in constructing silver(I) frameworks with HL. Solvent water molecules are key because they greatly affect the coordination geometries. Interestingly, although several Ni(II), Co(II) and Cd(II) compounds with HL have been prepared from solutions in water (Garribba *et al.*, 2006; Shang *et al.*,2007; Liu *et al.*, 2007; Ciurtin *et al.*, 2003; Ciurtin *et al.*, 2001; Ptasiewicz-Bak & Leciejewicz, 2000), such arrangement of different metal(II) coordination geometries induced by coordinated water molecules are not observed. This may be ascribed to the flexible and varied coordination geometries of silver atoms, *i.e.*, a metal-directing effect.

The one-dimensional double chains of the title compound are extended into a three-dimensional supramolecular architecture by nine O—H···O hydrogen bonds (Table 2). The detailed environments of the O—H···O interactions are represented in Fig. 2. Lattice water molecule O7 acts as hydrogen bond donors to lattice water molecule O8 forming binuclear water clusters. As shown in Fig. 3, O—H···O hydrogen bonds from carboxylate oxygen atoms and lattice water molecules link the chains into a two-dimensional supramolecular sheet: O8 acts as hydrogen donor to two carboxylate oxygen atoms (O3 and O4) forming a C_2_^2^(4) ring (Etter, 1990) and one carboxylate oxygen O1 of neighboring *L* ligands. O7 also acts as hydrogen bond acceptor to O5, O6 and acts as hydrogen bond donor to atom O3. Additionally O5 is also hydrogen bonded to O2 forming a strong O—H···O hydrogen bond, further consolidating the supramolecular sheet. Neighboring sheets are assembled into a three-dimensional supramolecular architecture by O6—H6B···O1 and O7—H7A···O8 hydrogen bonds (Fig. 3).

Besides classical O—H···O hydrogen bonds, also weaker non-classical C—H···O hydrogen bonds are observed (geometric details in Table 2), further extending the title compound into a three-dimensional supramolecular architecture. Additionally π–π stacking interactions are also be observed between two pyrazine groups with a distance of 3.643 (5) Å, which also help to stabilize the supramolecular architecture. The detailed environment of C—H···O interactions are also represented in Fig. 3.

### Experimental

{[Ag_2_(*L*)_2_(H_2_O)_2_]2H_2_O}_n_ (I) was prepared under the hydrotheraml conditions. AgNO_3 _(0.2 mmol), 5-methylpyrazine-2-carboxylic acid (0.2 mmol) was added into a 25 ml reaction vessel. the reaction vessel was then sealed and subsequently placed in an oven for 140 h at 120°C. The well shaped colorless block crystals suitable for single-crystal X-ray diffraction analysis can be obtained.

### Refinement

H atoms of water molecules were placed in calculated positions as riding atoms attached to non-riding atoms with O—H distances of 0.85 Å and with *U*_iso_(H) = 1.5*U*_eq_(O). H atoms bound to C atoms were placed geometrically and refined using a riding model with C(methyl)—H = 0.93 Å and C(phenyl)—H = 0.96 Å. The methyl H atoms were treated with AFIX137.

### Crystal data

**Table:**

[Ag_2_(C_6_H_5_N_2_O_2_)_2_(H_2_O)_2_]·2H_2_O	*Z* = 2
*M**_r_* = 562.04	*F*(000) = 552
Triclinic, *P*1	*D*_x_ = 2.185 Mg m^−^^3^
Hall symbol: -P 1	Mo *K*α radiation, λ = 0.71073 Å
*a* = 6.9481 (5) Å	Cell parameters from 2098 reflections
*b* = 10.1827 (8) Å	θ = 3.1–27.8°
*c* = 13.483 (1) Å	µ = 2.34 mm^−^^1^
α = 107.503 (1)°	*T* = 293 K
β = 100.185 (1)°	Block, colourless
γ = 103.164 (1)°	0.24 × 0.20 × 0.16 mm
*V* = 854.4 (1) Å^3^	

### Data collection

**Table:**

Bruker APEX CCD area-detector diffractometer	2982 independent reflections
Radiation source: fine-focus sealed tube	2518 reflections with *I* > 2σ(*I*)
graphite	*R*_int_ = 0.014
φ and ω scans	θ_max_ = 25.0°, θ_min_ = 1.6°
Absorption correction: multi-scan (ABSCOR; Higashi, 1995)	*h* = −8→7
*T*_min_ = 0.581, *T*_max_ = 0.698	*k* = −12→12
4422 measured reflections	*l* = −7→16

### Refinement

**Table:**

Refinement on *F*^2^	0 restraints
Least-squares matrix: full	H-atom parameters constrained
*R*[*F*^2^ > 2σ(*F*^2^)] = 0.027	*w* = 1/[σ^2^(*F*_o_^2^) + (0.0298*P*)^2^ + 0.2298*P*] where *P* = (*F*_o_^2^ + 2*F*_c_^2^)/3
*wR*(*F*^2^) = 0.071	(Δ/σ)_max_ = 0.001
*S* = 1.07	Δρ_max_ = 0.39 e Å^−^^3^
2982 reflections	Δρ_min_ = −0.51 e Å^−^^3^
236 parameters	

### Special details

**Table:**

Geometry. All e.s.d.'s (except the e.s.d. in the dihedral angle between two l.s. planes) are estimated using the full covariance matrix. The cell e.s.d.'s are taken into account individually in the estimation of e.s.d.'s in distances, angles and torsion angles; correlations between e.s.d.'s in cell parameters are only used when they are defined by crystal symmetry. An approximate (isotropic) treatment of cell e.s.d.'s is used for estimating e.s.d.'s involving l.s. planes.
Refinement. Refinement of *F*^2^ against ALL reflections. The weighted *R*-factor *wR* and goodness of fit *S* are based on *F*^2^, conventional *R*-factors *R* are based on *F*, with *F* set to zero for negative *F*^2^. The threshold expression of *F*^2^ > σ(*F*^2^) is used only for calculating *R*-factors(gt) *etc*. and is not relevant to the choice of reflections for refinement. *R*-factors based on *F*^2^ are statistically about twice as large as those based on *F*, and *R*- factors based on ALL data will be even larger.

### Fractional atomic coordinates and isotropic or equivalent isotropic displacement parameters (Å^2^)

**Table:**

	*x*	*y*	*z*	*U*_iso_*/*U*_eq_	
Ag1	0.51241 (5)	0.71236 (3)	0.76748 (2)	0.04603 (12)	
Ag2	0.92839 (4)	0.50359 (3)	1.21079 (2)	0.04547 (11)	
O1	0.6620 (4)	0.2358 (3)	0.8569 (2)	0.0435 (6)	
O2	0.8186 (4)	0.2942 (3)	1.0300 (2)	0.0440 (6)	
O3	0.2277 (4)	0.2659 (3)	0.5098 (2)	0.0475 (7)	
O4	0.3568 (4)	0.4346 (3)	0.6721 (2)	0.0508 (7)	
O5	0.1848 (4)	0.7679 (3)	0.7910 (2)	0.0525 (7)	
H5A	0.1404	0.8206	0.7600	0.079*	
H5B	0.1849	0.7866	0.8568	0.079*	
O6	0.7016 (5)	0.9584 (3)	0.7828 (3)	0.0646 (9)	
H6A	0.8065	0.9640	0.7582	0.097*	
H6B	0.6753	1.0373	0.8091	0.097*	
O7	0.9786 (4)	0.0754 (3)	0.3171 (2)	0.0489 (7)	
H7A	0.8716	0.0125	0.3142	0.073*	
H7B	1.0455	0.1352	0.3798	0.073*	
O8	0.3319 (4)	0.1221 (3)	0.6643 (3)	0.0627 (8)	
H8A	0.4339	0.1576	0.7196	0.094*	
H8B	0.3065	0.1766	0.6301	0.094*	
N1	0.6624 (4)	0.6564 (3)	0.9057 (2)	0.0309 (6)	
N2	0.8050 (4)	0.5719 (3)	1.0748 (2)	0.0282 (6)	
N3	0.3601 (4)	0.6478 (3)	0.5860 (2)	0.0315 (6)	
N4	0.1450 (4)	0.5712 (3)	0.3731 (2)	0.0325 (6)	
C1	0.6657 (5)	0.5205 (3)	0.8899 (3)	0.0283 (7)	
H1	0.6178	0.4531	0.8201	0.034*	
C2	0.7365 (4)	0.4767 (3)	0.9722 (3)	0.0268 (7)	
C3	0.8019 (5)	0.7062 (4)	1.0902 (3)	0.0327 (8)	
H3	0.8484	0.7734	1.1601	0.039*	
C4	0.7324 (5)	0.7511 (4)	1.0068 (3)	0.0308 (7)	
C5	0.7297 (6)	0.9034 (4)	1.0286 (3)	0.0481 (10)	
H5A'	0.6457	0.9094	0.9663	0.072*	
H5B'	0.6748	0.9339	1.0892	0.072*	
H5C'	0.8668	0.9649	1.0442	0.072*	
C6	0.7382 (5)	0.3213 (4)	0.9515 (3)	0.0301 (7)	
C7	0.3370 (5)	0.7465 (4)	0.5428 (3)	0.0348 (8)	
H7	0.3963	0.8436	0.5852	0.042*	
C8	0.2759 (5)	0.5087 (4)	0.5229 (3)	0.0290 (7)	
C9	0.1744 (5)	0.4725 (4)	0.4164 (3)	0.0323 (7)	
H9	0.1242	0.3755	0.3727	0.039*	
C10	0.2270 (5)	0.7099 (4)	0.4361 (3)	0.0325 (8)	
C11	0.1903 (6)	0.8224 (4)	0.3919 (3)	0.0446 (9)	
H11A	0.0462	0.8116	0.3739	0.067*	
H11B	0.2389	0.8117	0.3284	0.067*	
H11C	0.2623	0.9164	0.4451	0.067*	
C12	0.2901 (5)	0.3943 (4)	0.5726 (3)	0.0337 (8)	

### Atomic displacement parameters (Å^2^)

**Table:**

	*U*^11^	*U*^22^	*U*^33^	*U*^12^	*U*^13^	*U*^23^
Ag1	0.0615 (2)	0.04191 (18)	0.02887 (17)	0.01567 (14)	−0.00615 (14)	0.01461 (14)
Ag2	0.0587 (2)	0.04844 (19)	0.02474 (17)	0.01533 (15)	−0.00451 (14)	0.01602 (14)
O1	0.0578 (16)	0.0322 (13)	0.0318 (14)	0.0159 (12)	0.0002 (12)	0.0042 (12)
O2	0.0594 (16)	0.0437 (15)	0.0361 (15)	0.0211 (13)	0.0066 (13)	0.0235 (13)
O3	0.0637 (17)	0.0331 (15)	0.0403 (15)	0.0112 (13)	0.0052 (14)	0.0129 (13)
O4	0.0679 (17)	0.0483 (16)	0.0298 (15)	0.0125 (13)	−0.0072 (13)	0.0203 (13)
O5	0.0775 (19)	0.0463 (16)	0.0371 (15)	0.0244 (14)	0.0124 (14)	0.0168 (13)
O6	0.085 (2)	0.0323 (15)	0.085 (2)	0.0153 (14)	0.0464 (19)	0.0196 (16)
O7	0.0594 (16)	0.0432 (15)	0.0392 (16)	0.0134 (13)	0.0068 (13)	0.0128 (13)
O8	0.0576 (17)	0.0515 (17)	0.076 (2)	0.0076 (14)	−0.0009 (16)	0.0351 (17)
N1	0.0363 (15)	0.0281 (15)	0.0233 (15)	0.0079 (12)	−0.0021 (12)	0.0093 (12)
N2	0.0315 (14)	0.0321 (15)	0.0185 (14)	0.0094 (12)	0.0025 (12)	0.0078 (12)
N3	0.0347 (15)	0.0332 (16)	0.0236 (15)	0.0097 (12)	0.0001 (12)	0.0107 (13)
N4	0.0339 (15)	0.0387 (17)	0.0248 (15)	0.0108 (13)	0.0034 (12)	0.0137 (13)
C1	0.0310 (17)	0.0273 (17)	0.0220 (17)	0.0086 (14)	0.0012 (14)	0.0054 (14)
C2	0.0208 (15)	0.0322 (18)	0.0238 (17)	0.0060 (13)	0.0006 (13)	0.0097 (15)
C3	0.0385 (18)	0.0325 (18)	0.0203 (17)	0.0102 (15)	0.0018 (15)	0.0036 (15)
C4	0.0318 (17)	0.0305 (18)	0.0285 (18)	0.0096 (14)	0.0031 (15)	0.0109 (15)
C5	0.073 (3)	0.0265 (19)	0.036 (2)	0.0127 (18)	0.001 (2)	0.0071 (17)
C6	0.0303 (17)	0.0310 (18)	0.033 (2)	0.0109 (14)	0.0105 (15)	0.0143 (16)
C7	0.0382 (19)	0.0315 (18)	0.0309 (19)	0.0081 (15)	0.0041 (16)	0.0105 (16)
C8	0.0245 (16)	0.0384 (19)	0.0276 (18)	0.0127 (14)	0.0065 (14)	0.0143 (16)
C9	0.0357 (18)	0.0324 (18)	0.0274 (18)	0.0125 (15)	0.0046 (15)	0.0093 (15)
C10	0.0319 (17)	0.040 (2)	0.0323 (19)	0.0138 (15)	0.0096 (15)	0.0196 (17)
C11	0.057 (2)	0.042 (2)	0.039 (2)	0.0143 (18)	0.0074 (19)	0.0222 (19)
C12	0.0296 (17)	0.037 (2)	0.037 (2)	0.0119 (15)	0.0048 (15)	0.0170 (17)

### Geometric parameters (Å, °)

**Table:**

Ag1—N1	2.260 (3)	N2—C2	1.353 (4)
Ag1—N3	2.311 (3)	N3—C7	1.330 (4)
Ag1—O6	2.478 (3)	N3—C8	1.339 (4)
Ag1—O5	2.517 (3)	N4—C10	1.335 (4)
Ag1—O4	2.598 (3)	N4—C9	1.340 (4)
Ag2—N4^i^	2.233 (3)	N4—Ag2^iii^	2.233 (3)
Ag2—N2	2.250 (3)	C1—C2	1.369 (4)
Ag2—O2	2.558 (3)	C1—H1	0.9300
Ag2—O5^ii^	2.688 (3)	C2—C6	1.525 (4)
Ag2—O4^ii^	2.809 (3)	C3—C4	1.387 (5)
O1—C6	1.244 (4)	C3—H3	0.9300
O2—C6	1.244 (4)	C4—C5	1.495 (5)
O3—C12	1.249 (4)	C5—H5A'	0.9600
O4—C12	1.243 (4)	C5—H5B'	0.9600
O5—H5A	0.8500	C5—H5C'	0.9600
O5—H5B	0.8500	C7—C10	1.396 (5)
O6—H6A	0.8500	C7—H7	0.9300
O6—H6B	0.8500	C8—C9	1.378 (5)
O7—H7A	0.8501	C8—C12	1.521 (5)
O7—H7B	0.8501	C9—H9	0.9300
O8—H8A	0.8499	C10—C11	1.491 (5)
O8—H8B	0.8499	C11—H11A	0.9600
N1—C4	1.336 (4)	C11—H11B	0.9600
N1—C1	1.342 (4)	C11—H11C	0.9600
N2—C3	1.326 (4)		
			
N1—Ag1—N3	149.97 (10)	N2—C2—C6	118.5 (3)
N1—Ag1—O6	110.25 (10)	C1—C2—C6	121.6 (3)
N3—Ag1—O6	92.51 (10)	N2—C3—C4	123.0 (3)
N1—Ag1—O5	111.71 (9)	N2—C3—H3	118.5
N3—Ag1—O5	83.98 (9)	C4—C3—H3	118.5
O6—Ag1—O5	95.92 (9)	N1—C4—C3	119.7 (3)
N1—Ag1—O4	84.35 (9)	N1—C4—C5	119.3 (3)
N3—Ag1—O4	67.85 (9)	C3—C4—C5	120.9 (3)
O6—Ag1—O4	154.82 (9)	C4—C5—H5A'	109.5
O5—Ag1—O4	97.40 (9)	C4—C5—H5B'	109.5
N4^i^—Ag2—N2	146.43 (10)	H5A'—C5—H5B'	109.5
N4^i^—Ag2—O2	138.04 (9)	C4—C5—H5C'	109.5
N2—Ag2—O2	69.03 (9)	H5A'—C5—H5C'	109.5
C6—O2—Ag2	114.8 (2)	H5B'—C5—H5C'	109.5
C12—O4—Ag1	114.3 (2)	O2—C6—O1	127.0 (3)
Ag1—O5—H5A	119.7	O2—C6—C2	116.9 (3)
Ag1—O5—H5B	108.3	O1—C6—C2	116.1 (3)
H5A—O5—H5B	116.9	N3—C7—C10	122.5 (3)
Ag1—O6—H6A	115.2	N3—C7—H7	118.8
Ag1—O6—H6B	128.7	C10—C7—H7	118.8
H6A—O6—H6B	116.2	N3—C8—C9	119.8 (3)
H7A—O7—H7B	115.7	N3—C8—C12	118.5 (3)
H8A—O8—H8B	117.8	C9—C8—C12	121.7 (3)
C4—N1—C1	117.3 (3)	N4—C9—C8	122.8 (3)
C4—N1—Ag1	122.3 (2)	N4—C9—H9	118.6
C1—N1—Ag1	120.1 (2)	C8—C9—H9	118.6
C3—N2—C2	117.1 (3)	N4—C10—C7	119.7 (3)
C3—N2—Ag2	122.3 (2)	N4—C10—C11	118.8 (3)
C2—N2—Ag2	120.5 (2)	C7—C10—C11	121.4 (3)
C7—N3—C8	117.7 (3)	C10—C11—H11A	109.5
C7—N3—Ag1	121.3 (2)	C10—C11—H11B	109.5
C8—N3—Ag1	120.7 (2)	H11A—C11—H11B	109.5
C10—N4—C9	117.4 (3)	C10—C11—H11C	109.5
C10—N4—Ag2^iii^	121.7 (2)	H11A—C11—H11C	109.5
C9—N4—Ag2^iii^	120.3 (2)	H11B—C11—H11C	109.5
N1—C1—C2	122.9 (3)	O4—C12—O3	125.0 (3)
N1—C1—H1	118.6	O4—C12—C8	118.0 (3)
C2—C1—H1	118.6	O3—C12—C8	116.9 (3)
N2—C2—C1	119.9 (3)		
			
N4^i^—Ag2—O2—C6	−159.0 (2)	Ag2—N2—C3—C4	177.9 (2)
N2—Ag2—O2—C6	−3.8 (2)	C1—N1—C4—C3	−0.9 (5)
N1—Ag1—O4—C12	−162.1 (3)	Ag1—N1—C4—C3	172.7 (2)
N3—Ag1—O4—C12	6.3 (2)	C1—N1—C4—C5	−179.3 (3)
O6—Ag1—O4—C12	−34.7 (4)	Ag1—N1—C4—C5	−5.7 (4)
O5—Ag1—O4—C12	86.7 (2)	N2—C3—C4—N1	0.8 (5)
N3—Ag1—N1—C4	178.7 (2)	N2—C3—C4—C5	179.2 (3)
O6—Ag1—N1—C4	41.6 (3)	Ag2—O2—C6—O1	−175.6 (3)
O5—Ag1—N1—C4	−63.7 (3)	Ag2—O2—C6—C2	5.9 (3)
O4—Ag1—N1—C4	−159.5 (3)	N2—C2—C6—O2	−5.7 (4)
N3—Ag1—N1—C1	−7.9 (4)	C1—C2—C6—O2	175.1 (3)
O6—Ag1—N1—C1	−144.9 (2)	N2—C2—C6—O1	175.7 (3)
O5—Ag1—N1—C1	109.7 (2)	C1—C2—C6—O1	−3.6 (4)
O4—Ag1—N1—C1	14.0 (2)	C8—N3—C7—C10	1.0 (5)
N4^i^—Ag2—N2—C3	−27.5 (3)	Ag1—N3—C7—C10	−173.2 (2)
O2—Ag2—N2—C3	−177.1 (3)	C7—N3—C8—C9	1.7 (5)
N4^i^—Ag2—N2—C2	150.2 (2)	Ag1—N3—C8—C9	175.9 (2)
O2—Ag2—N2—C2	0.6 (2)	C7—N3—C8—C12	−176.5 (3)
N1—Ag1—N3—C7	−164.1 (2)	Ag1—N3—C8—C12	−2.3 (4)
O6—Ag1—N3—C7	−23.9 (3)	C10—N4—C9—C8	3.1 (5)
O5—Ag1—N3—C7	71.8 (3)	Ag2^iii^—N4—C9—C8	−167.9 (2)
O4—Ag1—N3—C7	172.4 (3)	N3—C8—C9—N4	−3.9 (5)
N1—Ag1—N3—C8	21.9 (3)	C12—C8—C9—N4	174.3 (3)
O6—Ag1—N3—C8	162.1 (2)	C9—N4—C10—C7	−0.4 (5)
O5—Ag1—N3—C8	−102.2 (2)	Ag2^iii^—N4—C10—C7	170.5 (2)
O4—Ag1—N3—C8	−1.7 (2)	C9—N4—C10—C11	−177.6 (3)
C4—N1—C1—C2	0.1 (5)	Ag2^iii^—N4—C10—C11	−6.7 (4)
Ag1—N1—C1—C2	−173.6 (2)	N3—C7—C10—N4	−1.6 (5)
C3—N2—C2—C1	−0.8 (4)	N3—C7—C10—C11	175.4 (3)
Ag2—N2—C2—C1	−178.7 (2)	Ag1—O4—C12—O3	172.6 (3)
C3—N2—C2—C6	179.9 (3)	Ag1—O4—C12—C8	−9.6 (4)
Ag2—N2—C2—C6	2.1 (4)	N3—C8—C12—O4	8.6 (5)
N1—C1—C2—N2	0.7 (5)	C9—C8—C12—O4	−169.5 (3)
N1—C1—C2—C6	180.0 (3)	N3—C8—C12—O3	−173.4 (3)
C2—N2—C3—C4	0.1 (5)	C9—C8—C12—O3	8.4 (5)

Symmetry codes: (i) *x*+1, *y*, *z*+1; (ii) −*x*+1, −*y*+1, −*z*+2; (iii) *x*−1, *y*, *z*−1.

### Hydrogen-bond geometry (Å, °)

**Table:**

*D*—H···*A*	*D*—H	H···*A*	*D*···*A*	*D*—H···*A*
C1—H1···O4	0.93	2.38	3.061 (4)	130
O5—H5B···O2^ii^	0.85	1.94	2.675 (4)	144
O5—H5A···O7^iv^	0.85	1.91	2.756 (4)	175
O6—H6A···O7^v^	0.85	2.00	2.828 (4)	166
O6—H6B···O1^vi^	0.85	1.96	2.794 (4)	168
O7—H7A···O8^vii^	0.85	1.86	2.698 (4)	168
O7—H7B···O3^viii^	0.85	1.87	2.712 (4)	171
O8—H8A···O1	0.85	2.02	2.867 (4)	176
O8—H8B···O3	0.85	2.13	2.965 (4)	166
O8—H8B···O4	0.85	2.44	3.116 (4)	137

Symmetry codes: (ii) −*x*+1, −*y*+1, −*z*+2; (iv) −*x*+1, −*y*+1, −*z*+1; (v) −*x*+2, −*y*+1, −*z*+1; (vi) *x*, *y*+1, *z*; (vii) −*x*+1, −*y*, −*z*+1; (viii) *x*+1, *y*, *z*.
